# Mammary Analogue Secretory Carcinoma of the Palate: Case Report and Review of the Literature

**DOI:** 10.1155/2019/7416302

**Published:** 2019-02-27

**Authors:** Christian Boliere, James Murphy, Mohammed Qaisi, Frances Manosca, Henry Fung

**Affiliations:** ^1^Division of Oral & Maxillofacial Surgery, John H. Stroger Hospital of Cook County, Chicago, IL, USA; ^2^Division of Plastic and Reconstructive Surgery, Rush University Medical Center, Chicago, IL, USA; ^3^Division of Otolaryngology, John H. Stroger Hospital of Cook County, Chicago, IL, USA; ^4^Department of Pathology, John H. Stroger Hospital of Cook County, Chicago, IL, USA

## Abstract

Mammary analogue secretory carcinoma (MASC) is a recently described salivary gland tumor, with a limited number of published reports. Less than three hundred cases have been reported in the literature and only 18 of these cases have been reported in minor palatal salivary glands, though publication bias is likely a factor. We present a case of a 57-year-old male who was diagnosed with MASC tumor presenting in a minor salivary gland and briefly review the current literature. MASC has a variety of histological features and different range of clinical behaviors. The histopathological diagnosis of MASC can be difficult, and the immunohistochemical profile of MASC is still being updated. The gold standard for MASC diagnosis is cytogenetics, with the majority having a translocation t(12;15)(p133;q25). Presently, there is no conclusive evidence that MASC should be treated differently than any other low-grade malignant salivary gland tumors, though high-grade transformation has been described.

## 1. Introduction

Mammary analogue secretory carcinoma (MASC) is a rare salivary gland neoplasm that was first reported in 2010 by Skalova et al. [1]. In the original report, 16 cases of salivary gland tumors were reexamined and were found to have similar histological and molecular features as breast secretory carcinoma. Breast secretory carcinoma is a slow-growing, low-grade ductal carcinoma which is a subtype of infiltrating ductal carcinoma of the breast. It occurs primarily in adolescent women [2]. Both MASC and breast secretory carcinoma are associated with translocation t(12;15)(p13;q25), which is a fusion of the ETV6 gene on chromosome 12 and the NTRK3 gene on chromosome 15. The fusion gene encodes a chimeric tyrosine kinase, which has potential transformation activity and plays a role in carcinogenesis [3]. This fusion has also been shown in other tumors including infantile fibrosarcoma, acute myeloid leukemia, and congenital mesoblastic nephroma [4]. Fluorescence in situ hybridization for the ETV6 rearrangement, with close to 99% of MASC tumors showing this mutation, is the gold standard to arrive at a definitive diagnosis of MASC [5]. The financial burden of this cytogenetic test can be prohibitive for some institutions, and there have been reports of cases not demonstrating *ETV6-NTRK3* fusion gene but were diagnosed as MASC based on the results of immunohistochemistry [6].

According to Khurram et al., immunohistochemistry can accurately diagnose MASC tumors [7]. Refinement of the immunohistochemical panel for the diagnosis of MASC could potentially completely reduce the cytogenic need in diagnosing MASC tumor [8]. To complicate matters, Mariano et al. recently reported that some cases of MASC, whose profile was NTRK3 split-negative and ETV6 split-positive, unknown (non-NTRK) genes appeared to fuse with ETV6 (ETV6-X fusion) [9].

A significant proportion of MASC tumors reported in the literature was cases that were reclassified when they were secondarily reviewed. MASC tumors have a wider morphological and immunohistochemical spectrum than previously recognized. Diagnosis and differentiation of MASC from other salivary gland tumor are aided by histological features and immunohistochemistry. MASC rarely shows perineural invasion or lymphovascular invasion, and necrosis is not typical [8]. MASC shows immunoreactivity for S100 and mammaglobin (70% of the time [9]) which are rarely positive in acinic cell carcinoma. DOG-1 is predominantly negative in MASC but usually positive in acinic cell carcinoma. Mammary analogue secretory carcinoma of the salivary glands is a lipid-rich tumor, and adipophilin can be valuable in its identification [10]. There is a multitude of markers which is beyond the scope of this paper.

Seventy percent of MASC tumors are found in the major salivary glands, predominantly the parotid. Less than a quarter of the cases arise from minor salivary glands within the oral cavity [3]. We present a case of a MASC tumor presenting in the hard palate. To our knowledge, this is one of the few case reports of palatal MASC, which was diagnosed by immunohistochemistry from an incisional biopsy from the hard palate, and prospectively definitively treated as a MASC tumor. Twelve cases of MASC on the hard palate and six on the soft palate have been reported in the literature worldwide to date [11]. The clinical course, presentation, and immunohistological findings are discussed.

## 2. Case Presentation

A 57-year-old male presented to the oral and maxillofacial surgery clinic at our institution with more than a 20-year history of a painless mass in the hard palate region. He elected to have the lesion evaluated due to its increase in size, although indolent. He reported no other symptoms.

His past medical history was significant for hypertension and hyperlipidemia. He denies any past surgical history, he was not taking any medications, and he has no known drug allergies. He presently denies any social history but admits to tobacco use for 15 years and stopped almost 20 years ago.

**Figure 1:**
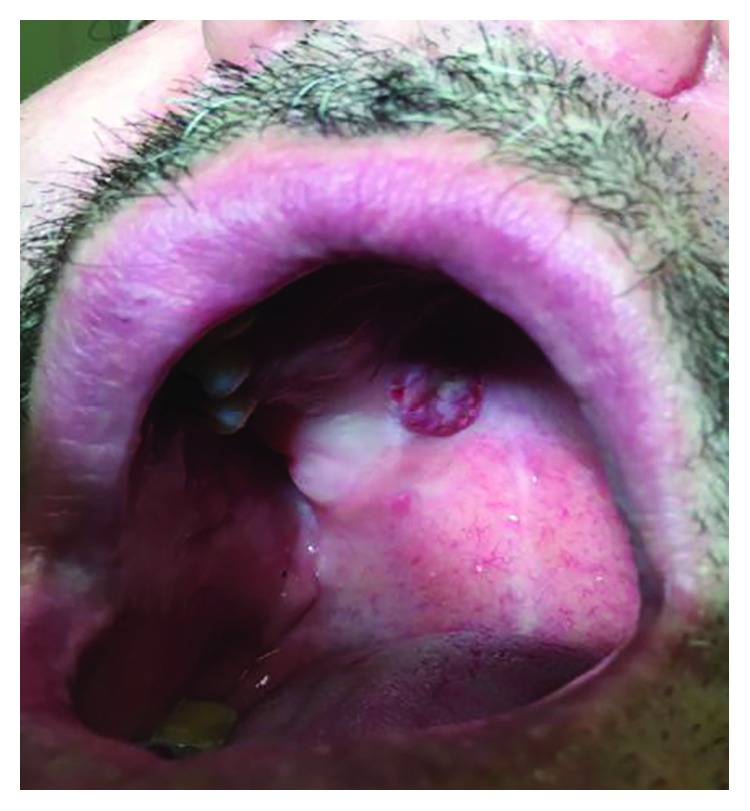
Preoperative image of MASC tumor right palate.

On examination, it was noted that the patient had a firm, erythematous, raised lesion with a central area of ulceration located at the junction of the hard and soft palate on the right side. The lesion was roughly 2.0 cm × 1.0 cm in size (Figure 1). There was no palpable lymphadenopathy on head and neck examination.

**Figure 2:**
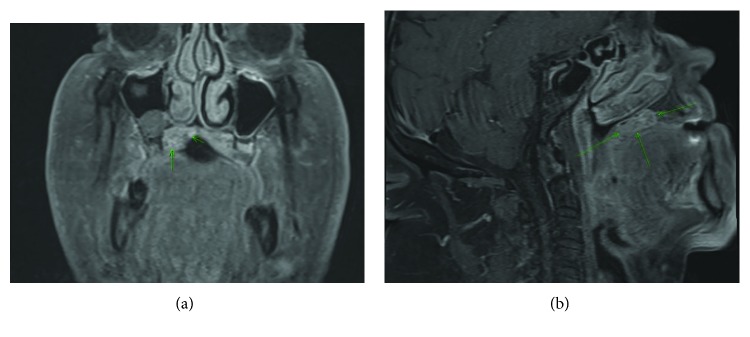
(a) MRI coronal T1image with MASC tumor. (b) MRI sagittal T1 image with MASC tumor.

Computed tomography (CT) and magnetic resonance imaging (MRI) showed a mass involving the right palate measuring 39 mm in the greatest dimension. The CT showed a focal area of bone thinning and focal dehiscence at the floor of the right maxillary sinus without evidence for frank tumor extension into the sinus (Figures 2(a) and 2(b)).

**Figure 3:**
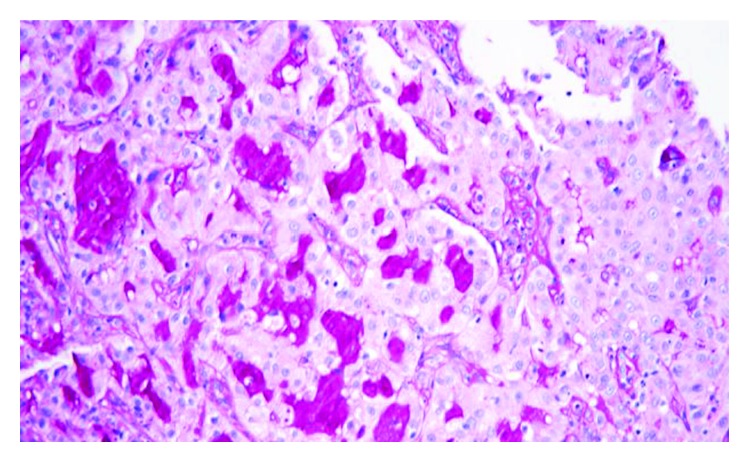
MASC tumor with PAS stain and diastase (magnification ×200).

**Figure 4:**
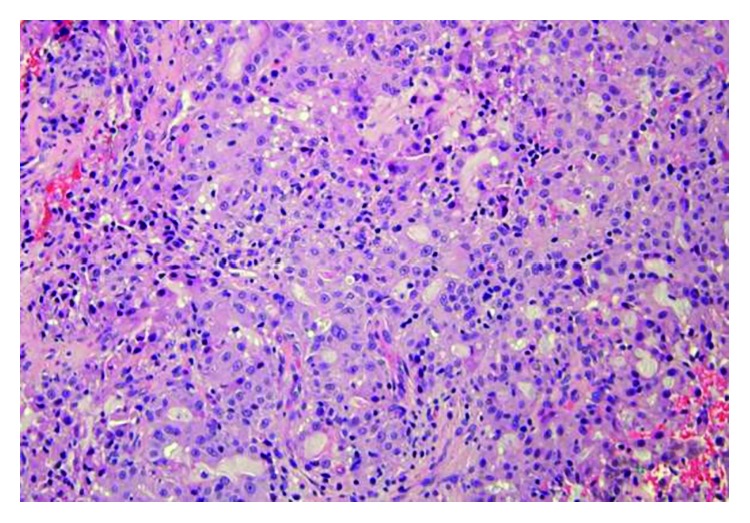
MASC tumor comprised of cells forming microcystic and glandular spaces containing eosinophilic homogenous material (H&E, magnification ×200).

**Figure 5:**
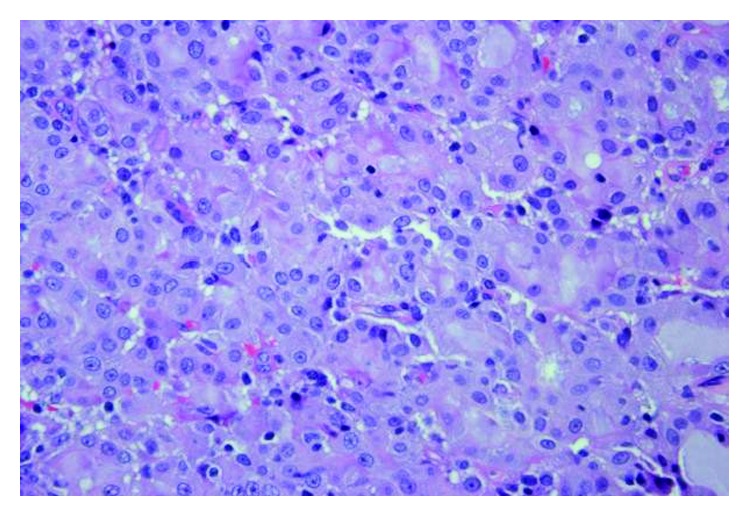
MASC tumor cells with eosinophilic granular cytoplasm and low-grade vesicular nuclei (H&E, magnification ×400).

**Figure 6:**
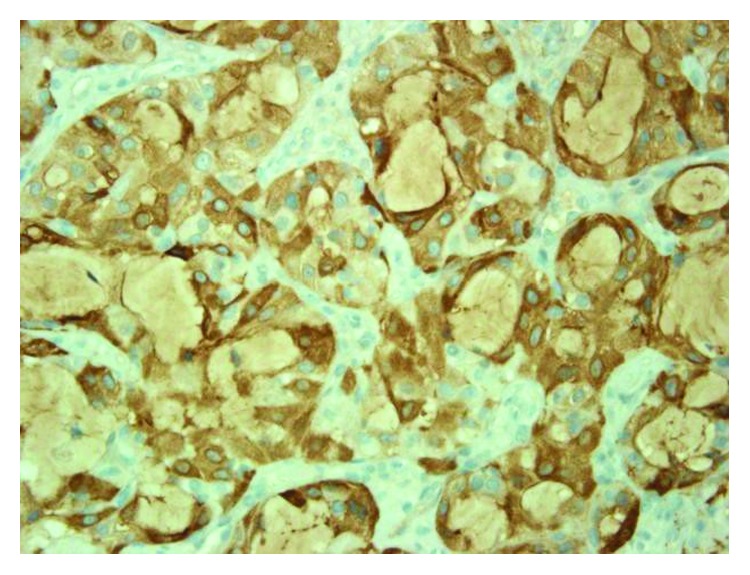
Mammaglobin highlights the neoplastic cells and secretions (magnification ×400).

**Figure 7:**
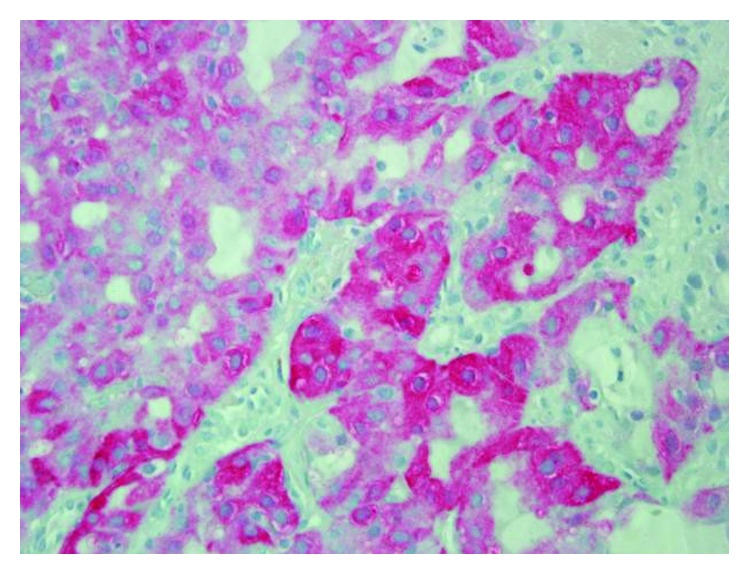
S100 protein staining is present in the tumor cells (magnification ×400).

An incisional biopsy was performed and sent for pathology. On hematoxylin and eosin stain, the tumor comprised of cells forming microcystic and glandular spaces containing eosinophilic homogenous material. The secretory material from the glandular spaces was noted to be periodic acid Schiff- (PAS-) positive and diastase-resistant (Figure 3). The tumor cells had eosinophilic granular cytoplasm with low-grade vesicular nuclei and visible nucleoli (Figures 4 and 5). There were rare mitotic figures, and scattered inflammatory cells were present. A broad front pattern of invasion was noted. Immunohistochemical stains showed positivity for cytokeratin 7, SMA, p53, and CK5/6. The tumor was also diffusely positive for both mammaglobin and S100, supportive for the diagnosis of MASC [7] (Figures 6 and 7). DOG-1 staining was negative. Cytogenic testing was not performed in this case.

**Figure 8:**
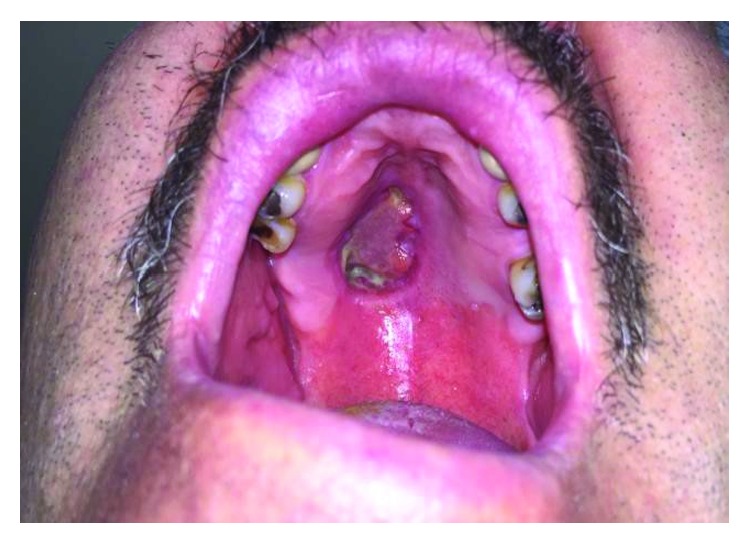
Postoperative image of surgical site healing by secondary intention.

Treatment consisted of wide local excision of the lesion with 1 cm margins and a right selective neck dissection levels 1-3. Hemostasis of the greater palatine neurovascular bundle was achieved with bipolar electrocautery and bone wax. The surgical site was allowed to heal by secondary intention with granulation tissue (Figure 8).

A total of 42 lymph nodes were harvested and all lymph nodes were negative. No lymphovascular and no perineural invasion was noted. All margins were negative with the closest margins at 0.5 mm. The final pathologic diagnosis was MASC with the tumor size measuring 1 × 0.7 × 0.6 cm. Given the negative margins and the lack of consensus regarding the utility of adjuvant therapy in this disease entity, no further treatment was administered.

**Figure 9:**
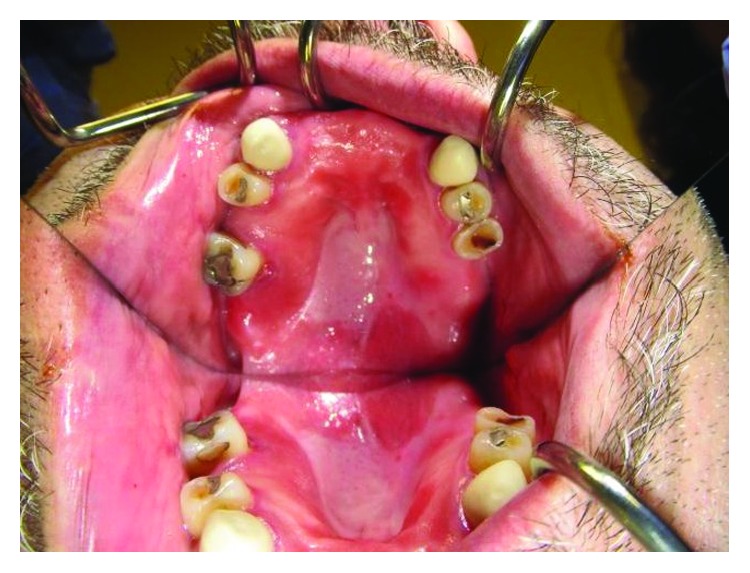
Surgical site at 36 months. No evidence of recurrence.

Patient's postoperative course was uneventful, although a biopsy was done 6 months later which showed no evidence of disease. At his most recent follow-up, 36 months after surgery, the patient was free of tumor (Figure 9). The patient had no neurologic deficit. He had no range of motion issues with his shoulder, however complained of slight numbness over his right shoulder. He denied any change in speech or swallowing issues. The nasal mucosa was preserved; levator muscles and palatal muscles were preserved. The hard palate site healed well by secondary intention.

## 3. Discussion

MASC involves the parotid gland in approximately 70% of cases. Less than 25% of MASC tumors have been reported in minor salivary glands, at sites including the palate, buccal mucosa, base of the tongue, and lips [12]. There has also been a case report on a lymph node with MASC of unknown primary origin [10].

The average age of patients presenting with MASC is in the fourth or fifth decade. The tumor is very rare in children and adolescents, but few cases have been reported [13]. MASC has been reported to have a slight male predilection [9], but no racial predisposition has been documented. The disease typically follows an indolent course. The most common presentation is a slowly enlarging and painless nodule, often detected incidentally on physical examination [12]. Our case presented with a similar description. There has been a report of one patient with facial paralysis from a bulky parotid gland MASC tumor.

The exact incidence of MASC is unknown. It is reported that MASC accounts for <0.3% of all salivary gland tumors [14]. Luk et al. and Majewska et al. reported that MASC makes up 4 and 4.5%, respectively, of malignant salivary gland disease processes [15]. The percentage of MASC tumors involving the minor salivary glands is unknown. In the most recent update, there has been a total of 279 MASC cases reported in the literature, and 68 (24%) cases of MASC reported in minor salivary glands [11].

In the current case, polymorphous low-grade adenocarcinoma (PLGA) was ruled out based on histopathological features including a well-defined broad front invasive pattern seen in this case as opposed to an infiltrative cord-like one typical in PLGA [16]. The presence of microcystic areas with abundant vacuolated colloid-like PAS-positive secretory material within the microcystic spaces, low-grade vesicular nuclei with a distinctive centrally located small nucleolus, the lack of perineural invasion, and the diffuse pattern of staining seen with S100 protein and mammaglobin were further features which support the diagnosis of MASC. Further histopathological evidence supporting the diagnosis of MASC was the fact that the luminal secretions were PAS-positive and diastase-resistant, and cytoplasmic granules were not present. Bishop et al. reported [17] that approximately 80% of extra parotid acinic cell carcinoma needed to be reclassified as MASC on the basis of an ETV6 translocation together with strong staining for S100 and mammaglobin. Pinto et al. reported in their study that 3 out of 6 MASC tumors were initially classified as acinic cell carcinoma [18]. In addition to acinic cell carcinoma, other cases of cystadenocarcinoma, mucoepidermoid carcinoma, or signet ring adenocarcinoma were reclassified as MASC [19]. Griffith et al. prospectively diagnosed a case of MASC based on cytogenetic study of a parotid mass fine needle aspiration (FNA), which was subsequently confirmed on final pathology [20]. The use of FNA in the diagnosis of MASC is still of questionable value [21]. Takeda et al. state that MASC cannot be diagnosed only from cytology alone [22]. Histopathological findings of MASC overlap with other salivary gland tumors, most commonly acinic cell carcinoma and adenocarcinoma not otherwise specified [12]. The histologic findings of MASC can include solid, microcystic, tubular, and papillary-cystic patterns in varying proportions. The case described in this report presented predominantly with microcystic features. MASC usually shows evidence of mucin production, a feature not seen in acinic cell carcinoma [23]. In MASC, the cells show vacuolated or foamy cytoplasm devoid of basophilic coarse zymogen granules, which may histologically resemble zymogen-poor acinic cell carcinoma. Many histologic findings seen in MASC and other salivary gland tumors overlap; therefore, the use of ancillary studies to aid in the distinction among the entities is important. New cytomorphology features were recently added to those of the original description though their clinical usefulness has not been studied extensively yet [9].

There is no data in the literature showing a difference in clinical behavior or rates of regional metastasis between minor and major salivary gland MASC. It is not uncommon for MASC to metastasize to regional lymph nodes. In one study, the rates of lymph node metastasis for MASC and acinic cell carcinoma were 17.6% (6/34) and 7.9% (3/38), respectively, with a mean disease-free survival, including death and recurrence, for MASC and acinic cell carcinoma being 92 and 121 months, respectively [24]. Although limited in numbers, other studies have explored treatment outcomes and prognosis of MASC and acinic cell carcinoma and concluded they can be treated similarly [25]. With regard to treatment, neck dissection is presently a surgeon preference based on clinical, radiological, and histological parameters. Sethi et al. reviewed 86 patients with MASC and reported 21 patients underwent neck dissections, 17 patients received postoperative radiotherapy (PORT), and 2 patients received PORT and chemotherapy (agents unspecified). No reported patients have received RT without prior surgical resection [12]. The rate of occult nodal metastasis for MASC is not well documented and we possibly need more reported cases, but one study reported that 4 out of 18 patients treated with neck dissections at the University of Pittsburg had nodal involvement [25].

MASC usually has an overall favorable prognosis; however, high-grade transformation has been described which results in a more aggressive tumor [14]. Stevens et al. noted high-grade transformation in 3/100 MASC cases reviewed [26]. Currently, MASC tumors with necrosis [24] tend to demonstrate a more aggressive nature and ultimately a poorer prognosis. Sethi et al. reported that of 91 cases of MASC documented at the time of writing, only 4 cases of death from the disease were reported, although survival data were variably reported, and follow-up was minimal.

## 4. Conclusion

We encountered a MASC tumor presenting in the region of the hard palate. The patient underwent complete excision of the lesion and a selective neck dissection without adjuvant therapy. No local recurrence or metastatic disease has been detected during a follow-up period of 36 months. This patient, with a prospectively diagnosed and treated MASC in the hard palate, is presented to add to the existing limited body of literature regarding this entity. The diagnosis in this case was based on the morphology with supporting S100 protein/mammaglobin immunoreactivity. Due to the histopathological findings, absence of unusual morphology, and the immunohistochemical profile, the financial burden associated with cytogenetic analysis to diagnose MASC was determined to be unnecessary. To the best of our knowledge, this is one of the few cases to report this method to prospectively diagnose a hard palate MASC tumor. This is an important finding for modern fiscally conservative medical systems to minimize escalating costs.
